# Reconstructing communities in cluster trials?

**DOI:** 10.1186/s13063-016-1284-6

**Published:** 2016-03-28

**Authors:** Sapfo Lignou, Sushmita Das, Jigna Mistry, Glyn Alcock, Neena Shah More, David Osrin, Sarah J. L. Edwards

**Affiliations:** University College London, London, UK; Society for Nutrition, Education and Health Action (SNEHA), Urban Health Centre, 60 Feet Road, Dharavi, Mumbai, 400017 Maharashtra India; Institute for Global Health, Institute of Child Health, 30 Guilford Street, London, WC1N 1EH UK

**Keywords:** Clusters, Community, CRTs, Community engagement, Mumbai, India

## Abstract

**Background:**

There is growing interest in the ethics of cluster trials, but no literature on the uncertainties in defining communities in relation to the scientific notion of the cluster in collaborative biomedical research.

**Methods:**

The views of participants in a community-based cluster randomised trial (CRT) in Mumbai, India, were solicited regarding their understanding and views on community. We conducted two focus group discussions with local residents and 20 semi-structured interviews with different respondent groups. On average, ten participants took part in each focus group, most of them women aged 18–55. We conducted semi-structured interviews with ten residents (nine women and one man) lasting approximately an hour each and seven individuals (five men and two women) identified by residents as local leaders or decision-makers. In addition, we interviewed two Municipal Corporators (locally elected government officials involved in urban planning and development) and one representative of a political party located in a slum community.

**Results:**

Residents’ sense of community largely matched the scientific notion of the cluster, defined by the investigators as a geographic area, but their perceived needs were not entirely met by the trial.

**Conclusion:**

We examined whether the possibility of a conceptual mismatch between ‘clusters’ and ‘communities’ is likely to have methodological implications for a study or to lead to potential social disharmony because of the research interventions, arguing that it is important to take social factors into account as well as statistical efficiency when choosing the size and type of clusters and designing a trial. One method of informing such a design would be to use existing forums for community engagement to explore individuals’ primary sense of community or social group and, where possible, to fit clusters around them.

**Trial registration:**

ISRCTN Register: ISRCTN56183183 Clinical Trials Registry of India: CTRI/2012/09/003004.

## Background

Cluster randomized trials (CRTs) have become an increasingly important tool in health research. However, the substantial methodological differences between cluster and individually randomized trials have ethical implications for protecting the rights and interests of the individuals within clusters. While many of these issues have been explored in the literature, some outstanding issues remain and are not addressed in current guidelines, including the recent Ottawa Statement. Moreover, the principle of respect for communities and practices such as community engagement have become important ethical requirements for the empowerment of participant communities and the protection of group interests in international collaborative research, as well as for enhancing the quality of research, in both international guidelines and the bioethics literature [1]. For instance, in the recent Ottawa Statement it is recommended that cluster consultation may ensure that the cluster randomized trial addresses local health needs and is conducted in accord with local values and customs [2]. However, there is not much guidance on a clear definition of ‘community’ and thus on useful direction for conducting community engagement or community consultation. As a result, researchers have employed a variety of definitions of community (external definitions), and utilized different practices and procedures, to secure the ethical conduct of their research [3].

The choice of clusters to recruit into trials may be influenced by a number of factors, including ease of recruitment and type of intervention to be evaluated. Clusters usually have geographical boundaries, although this is not always necessary. For statistical efficiency, it is important to keep the size of the cluster, in terms of participants, as small as is feasible.

The importance of communities and their protection in strengthening the ethics of international collaborative research is increasingly highlighted [4], but there has been debate about the meaning of the term ‘community’ and its specific normative significance. As a result, various definitions of community have been employed by researchers (for instance, in public health programmes and policy, community is where prevention and intervention take place [5]), and different practices have been used to consult or engage communities [3, 6, 7]. In general, community theorists agree that community indicates ‘a sense of belonging together’ [3], and may refer to a group of people with common characteristics, such as race, religion, profession or living in the same locality [3, 8-11].

There are often important differences between internal and external definitions of community: the way in which the members of a community define it and the way in which it is defined by others [12]. This dissonance between health investigators and researched communities can lead to dispute [13]. As Marsh et al. noted, in international collaborative research, definitions of community are usually made externally, based on the aims and context of a study (involving, for instance, groups of people with a certain disease or risk factor, those served by a particular health facility, living in the same geographical locality or having a legitimately elected leadership [3, 14–21]). People’s definitions of community have rarely been explored [3, 20].

Previous studies have shown that participants’ definitions of community do not necessarily coincide with those used by scientists. For instance, in a community consultation for emergency research, the authors found that researchers considered 'PAD Trial community' to mean persons of a specific age, those with a potential for cardiac arrests and within a geographically defined region (building or unit), while participants had a different view of their community [3]. Researchers in a recent vaccine trial found that participation established mechanisms for information sharing and created relationships between participants, but excluded other members of the same village [14]. Many discussions on protection of vulnerable groups in health research, and on guidelines for protection of indigenous communities in genomic research, have also been based on the fact that individuals’ rights and interests had been violated in the past by involuntary consideration of members of the groups studied [13].

A CRT involving informal settlements (slums) in Mumbai, India, presented us with an opportunity to consider some of these issues specifically in relation to cluster trials. Our objective was to examine the uncertainties in defining communities (by taking into account that there is a variety of definitions of community) in relation to the notion of the scientific cluster (taking into account that clusters are defined by methodological and practical concerns of the research proposal). We aimed to inform the idea of community in CRTs by developing an understanding of participants’ definition of community and the factors that help shape their views. We investigated whether residents’ sense of community matched the scientific notion of the cluster, defined by the investigators as a geographic area. We considered whether the possibility of mismatch was likely to have methodological implications for the study (beyond a simple statistical adjustment traditionally called the intra-cluster correlation coefficient, ICC), as well as present ethical challenges such as stigmatization of vulnerable groups, potential social disharmony because of the interventions in the study and political difficulties for any cluster representative. If there were differences between scientific and lay views, a cluster trial might create social and political conflicts by artificially dividing pre-existing communities or by forcing together different factions in the same cluster and offering interventions and shared resources only through coerced collaboration.

In examining participants’ idea of community, we wanted to explore how, in practice, to determine the meaning of the concept of ‘community’ in different contexts (that is, participants’ particular conceptions of community in different studies being conducted and among different populations with different conceptions of community) and how researchers should take into account these conceptions in determining clusters in their studies.

## Methods

### Setting

Half of Mumbai’s 12.5 million inhabitants live in informal settlements (Officer of the Registrar General & Census Commissioner, Director of Census Operations Maharashtra: *Census of India 2011. Provisional population totals: paper 1 of 2011: Maharashtra.* New Delhi: Ministry of Home Affairs, Government of India; 2011). Slum dwellers are worse off than those living in more conventional housing with respect to most health, nutrition and population indicators. About one-fifth of slum homes have a private toilet, 31 % of residents have completed 10 years of education, and the total fertility rate is below the replacement threshold at 1.9 (Government of India Ministry of Health and Family Welfare: National Family Health Survey, India (NFHS-3 2005–06), and Mumbai: International Institute for Population Sciences; 2007). The city is divided into 24 municipal wards for administrative convenience. Of these, M East ward has the lowest literacy rate (66 %), the highest infant mortality rate (66 per 1,000), the poorest human development ranking (0.05) and a high proportion of slum settlements. L ward is ranked second lowest, with a human development index of 0.29, and both of these vulnerable wards have large migrant populations, low and insecure levels of livelihood activity, large-scale unauthorized housing and poor education and health facilities.

For these reasons, M East and L wards were selected for a CRT of an intervention to improve the health and nutrition of women and children through community resource centres [22]. The trial involves 40 informal settlements, each having approximately 600 households: 20 areas were allocated to have community resource centres and 20 acted as controls. Allocation was done in three blocks of 12, 12 and 16 communities. Resource centres were set up in three phases, of 6, 6 and 8 centres, respectively, with six-month intervals between the start of each phase.^1^ The centres were set up to act as bases for collection and dissemination of health information, provision of services and referral of individuals and families to appropriate services. The effects of the intervention will be evaluated against indicators of maternal health and infant feeding, women’s reproductive health, violence against women and children and childhood nutrition. Outcomes will be compared with those in the 20 control settlements.

### Data collection

Within the trial activities, we collected qualitative data to understand participants’ perceptions of community and the factors that shaped their views. Participants were also asked about preferred methods of consultation, representation and their satisfaction with methods actually employed in the study. Answers to these questions may have practical implications for cluster trials, but will be the focus of another paper due to the constraints of word length and the importance of the issues. We recruited participants from across several intervention and control clusters in order to involve residents with different socio-economic and demographic backgrounds. Data collection took place between August and October 2012. Separate semi-structured questionnaires were designed to guide the focus group discussions and individual interviews. They were developed iteratively through conceptual discussions between SL, SJLE, DO, SD and GA, a multidisciplinary team with backgrounds in medicine, research ethics, social sciences and both quantitative and qualitative research methods. Discussions focused on the general content, topics of interest and the structure of individual questions. SL and JM (a local translator/research assistant conversant with the study objectives and underlying concepts) piloted and refined the questionnaires to familiarize themselves with the meaning and flow of questions and to ensure that translations were comprehensible. The researcher/translator felt more comfortable orally translating from questionnaires in English to local languages during data collection.

The questionnaires were divided into broad sections on respondent background, understanding of community, community health, perceptions of risk (associated with participation in a cluster trial), representation (by decision-makers) and understanding and acceptance of community-based research. The section on community was designed to explore how respondents understood and described the concept and meaning of ‘community’, based on their experiential knowledge of living in a Mumbai informal settlement. Given their complexity, discussions about uncertainty and risk associated with participating in community-based research trials were developed more in interviews with decision-makers and group discussions with residents than in individual interviews with residents.

The aim of the focus group discussions was to obtain a broad sense of residents’ understandings and views on community, health and community-based research, and the individual interviews to explore themes in more detail, drawing upon individuals’ experiences of inclusion in the cluster trial. Potential respondents were identified by community organizers, word of mouth or casually during fieldwork in their communities. SL and JM conducted all group discussions and individual interviews. The focus groups were held in SNEHA community centres, the in-depth interviews with residents in their homes, and the interviews with decision-makers in their homes or offices.

We conducted two focus group discussions with local residents and 20 semi-structured interviews with different respondent groups. On average, ten participants took part in each focus group, most of them women aged 18–55. We conducted semi-structured interviews with ten residents (nine women and one man) lasting approximately one hour each, and seven individuals (five men and two women) identified by residents as local leaders or decision-makers. In addition, we interviewed two Municipal Corporators (locally elected government officials involved in urban planning and development) and one representative of a political party located in a slum community. We obtained informed consent from each participant prior to interview. Interviewers explained the purpose and scope of the study and gave participants an information sheet and consent form in Hindi. We sought verbal consent because the interviews did not include sensitive issues, confidentiality was assured and participants favoured it. Any identifying information was anonymized in interview transcripts. Data collection ceased once it was felt that no new themes were emerging or that concepts and categories appeared to be sufficiently explored.

Each interview was audio-recorded and subsequently translated and transcribed in English. Care was taken to minimize misinterpretation from changes in meaning or bias during the translation process [23]. Interview transcripts included a paragraph describing the background, setting and process of the data collection activity. GA reviewed initial transcripts to check for quality and suggest improvements in data collection.

### Data analysis

SL reviewed individual interview transcripts and discussed them with SJLE in order to identify key emergent themes. These were used to inform subsequent data collection and in the development of early analysis. Thematic data were entered into a spreadsheet in Microsoft Office Excel and organized into columns of cases and rows of transcribed data excerpts. Given the relatively small number of participants, data were manually analysed using a thematic analysis approach [24]. After numerous further reviews of the transcripts by SL, the findings were discussed with DO, SD and GA. These discussions encouraged greater interrogation of the data from different conceptual and philosophical positions. Several drafts of the analysis were written by SL and SJLE, providing the opportunity to further refine the themes and provide a fairly rigorous interpretative framework with which to conceptualize and present the findings.

### Ethical approval

The study was approved by the Multi-institutional Ethics Committee of the Anusandhan Trust in March 2010.

## Results

Many respondents did not immediately identify with the term ‘community’ and some struggled to understand the questions: When we talk about ‘community’ how do you think of it? What comes to your mind? As a result, the interviewer sometimes prompted the respondent with the idea of community as being geographically based, which invited a closed choice. This may have suggested that respondents were unsure of what constituted their community or that the idea of community did not always sit comfortably with those who live in mixed ethnic and racial groups where there might have been a history of discord. In addition to notions of community, which related directly to the scientific idea of a geographically bound cluster, respondents were asked specifically about community relations to help interpret their uncertainty, and for any additional functional conceptions of community.

The transcripts yielded four main themes describing notions of community: participants living in a locality; social cohesion; shared problems or projects; and the moral status of groups. While these ideas offered a mixed definition of community, the responses at least suggested a common denominator: a shared identity with others. On top of this idea, other different and sometimes inconsistent thoughts on community seemed to emerge, suggesting that respondents were using the term flexibly to suit their different needs and preconceptions.

### Community as people living in a locality

Almost all respondents included a geographical element in their definitions. “… I consider everyone to be my community. The people who stay around, who stay in our area, they are only our community people” (resident, intervention area). Most defined their community as a group of people living in the same area, which conveyed a shared sense of ‘localness’ from their immediate environment:

*“Community is one … that which comprises local people - that’s community” (resident, intervention area)*

*“For me community is basically my surrounding, my people around me; that’s community for me” (corporator, area unknown*^2^*)*

Everyone said that community comprised their neighbours, regardless of religious, ethnic or caste differences. These responses were spontaneous and suggest that the idea of living in a ‘mixed’ community required explanation for researchers. For example, one male resident in a control area said, “… here there are Maharashtrians, Mohammedans and people of other caste, religion and ethnicity. It’s a mixed community here.” Interestingly, few respondents included religion alone as the determining factor. Many thought that it was important to give explicit acceptance to the idea that people with different religious beliefs could stay together in one place. The double negative in a response such as “we are *not* from *different* communities” may have reflected a degree of ambivalence or the need to express a socially acceptable view:

*“… be it Hindu, Muslim, Christian, we don’t feel that we are from different communities. We all stay together, we become part of one community. Wherever we meet, we are known as being people from Z. So, we trust the community that stays here and stays together” (resident, area unknown)*

As with religion, so with caste. Respondents explicitly and spontaneously rejected the view that community equated to caste, a view which they recognized might be common:

*“These caste-caste people say that this is my community … It is not … that I will consider people of my caste to be my community. Everyone is my community” (resident, intervention area)*

Here, community is broader than ethnicity or caste, more embracing of society. Given that all respondents lived in informal settlements, the explicit desire to include people from other castes is interesting and may convey a sense of social aspiration by identifying, or even a wish to project a sense of social responsibility onto those more fortunate by claiming a common bond. It seemed acceptable, however, to identify with a place of origin. Some identified their communities as from Uttar Pradesh, some from Gujarat — a solidarity that was often accompanied by language differences — and some in terms of long duration of residence.

The boundaries of the geographical community were closer for some than for others, especially when additionally determined by social relationships:

*“… the thing is, our area is divided … This way if you come here and ask us, then we will tell you … these two roads are divided. So in between these two roads, the 10 and 11 Road is there. If you come with people from there and then if you want some information, then why will we give you?” (resident, intervention area)*

The respondent, who lived in Road 13, said that she had refused to participate in a study in which the researchers were accompanied by people from Roads 11 and 12. Such a strong affiliation with a narrow geographical area associated with single lanes may have been based on the respondent’s ethnicity or religion as Maharashtrian Hindu in a predominantly Muslim area in which many had migrated from northern states.

### Community in terms of social cohesion

A few respondents included family in their responses about community. “My community is my children … my family … my own. That’s it” (resident, intervention area). The relative importance of different members of a social network may be reflected in the order in which the respondent listed them: children first, family second, and own people last, as if in concentric circles. The contrary view was that neighbours were part of the family:

*“It’s a mixed community here. We think of everyone as being our brother and sister, relative, and accordingly we stay. Here, I do not have any relatives. They are all at different-different places. But the people of this mohalla (area), they are all my relatives” (resident, control area)*

*“The whole area, lane, is my friend. And relatives, no one is there” (resident, intervention area)*

As well as conveying a feeling of closeness by identifying community with family, others emphasized the quality or harmonious nature of relationships based on communication and a common language, and gauged their perceived intimacy as analogous to family relations or to friends.

*“[Our] Relations are very good with each other. For example, I met you now, so now you have become my Madam (denoting respect towards the female researcher). Now when we start talking gradually, then you will call me didi (sister) or I will call you didi … So that way we have the same relation in the area. We call some people chacha (paternal uncle), mama (maternal uncle), some we call khala (maternal aunt), some we call buwa (paternal aunt). This way the relation is like home … Being related as friends is the best thing …” (resident, intervention area).*

This view was not ubiquitous. Some respondents said that residents of the same area had good, but not intimate, relations with each other, and could not be described as friends. Nevertheless, most respondents said that they lived in a united area where all residents had good relations with each other, regardless of their religious or caste differences, an avowal that we have already mentioned. “We maintain harmony with every religion. For us, this is our religion and the other religion is not [our religion]. We maintain relations with people of other religions too” (resident, area unknown). Maintaining relations might sound a little less than heartfelt and the sweeping generalization to every religion might suggest reluctance to cause disquiet. One respondent used the metaphor of sharing a meal to convey the closeness of community relations and the social ritual of eating: “… Everyone … used to sit and eat from the same plate. The Hindus, Christians everyone used to sit and eat from the same plate” (resident, area unknown).

For some, the good relations between groups in their community contrasted with those in other areas.

*“Now that everyone stays like one; like one. Now once again they had come, everyone … Everyone stays like one here … Everyone treats us nicely. The people in villages are different. Here no one will think that way” (resident, intervention area).*

This is another example of defining one’s community in terms of what it is not. In this case, urban life was compared with rural life. Others compared long-term residents with newcomers. “But in our area, it was we Hindu, Muslim, people belonging to all the religions that are there, we united so that no person from outside can come inside” (resident, control area).

Some respondents, however, believed that their area was not united. Distinctions were made between established residents and recent arrivals, with some effort to ascribe blame for deterioration in quality of life:

*“The Muslims who have come from UP, Bihar, Bangladesh, they have made it very dirty. In this last five years this area has become completely dirty. But the Muslims who have come from Bangladesh, because of them there have been incidences of rape on small girls. Since then here the environment has become bad… since the last 10 years; ever since the Samajwadi Party has come to power, Z (president of Samajwadi Party) … since then the gangsterism … lootmaar (vandalism)” (resident, area unknown).*

Violence was mentioned and, again, blamed on another group, with the emphasis on the reasonableness of the respondent’s position:

*“Now the children … of this new, new generation, even for small matters they indulge in physical violence. But the old residents who are there, we first make them understand. If they don’t get convinced then even we … the thing is, first of all we are not the kind of people who will indulge in physical violence. We just directly complain, dial the number (to police)” (resident, control area).*

### Community in terms of shared problems or projects

Some respondents said that being a member of a community meant helping neighbours when they were in need, especially when there was no immediate family living nearby. “I don’t have relatives here, but if something happens to me now, my entire house would be flooded with people” (resident, intervention area). Less typical responses included helping each other as a corollary of being related like family: “As far as the community is concerned, where we stay, all the local people, we stay like brothers. If they face some problem or if we face some problem then everyone will work together” (resident, intervention area).

Reflecting a view of community in terms of sharing health resources (or in terms of the collective need for a shared resource), many respondents mentioned the lack of a local government hospital, particularly for maternity services.

*“At least a hospital should be there. Pregnant women who are there, in Govandi that is there, the nursing home. When it is time to give birth to a child, that time the woman is struggling between life and death … Even the child’s life is (at risk), and regarding health, everyone here … thinks. Because when they give birth to kids, it is also important to think about them, isn’t it?” (resident, intervention area).*

Environmental conditions represented a shared experience that might draw people together and support their notion of community. Water supply — or the lack of it — was a common shared burden: “…the *nagar sevika* (corporator) here said water will be provided. The pipeline has been dug; pipes have been laid but only for show, to devour money” (resident, area unknown).

*“If you see the entire area surrounding … the dirt that falls out of that vehicle keeps falling on the road. In that, our kids play. In that, our women walk. And in that, we have to walk. That is not something less; it is a big bundle of diseases that is given to Z by the Municipal Corporation. Now, here the people seek employment. (Suppose) someone runs a welding factory here. Now if that vehicle has to be welded then its entire dirt will fall at that place. We cannot stop him. Because it is his employment, we cannot say no to him. In a way, he is helpless and we too are helpless. Because he has to earn his living, we suffer from diseases. Everyone staying in this area has to tolerate. So, all these things that are there, meaning this pollution, such big pollution, why is it in Z only? There are many things that one can say…” (gatekeeper, area unknown).*

*“So here there are many poor people. Since it is a slum area it is a very poor locality. So here there are many diseases … that are usually prevalent. Now if I open this (window) so much smell… will come from this dumping ground. That staying here … is difficult … difficult it is. Now here there are many diseases like TB, malaria, typhoid … Now mainly here there is the smell, dumping ground is there; biomedical this has started…. the entire Mumbai’s filth is there. The children here are not safe. Here there is smell, there is … How can the children be safe? [There is] Always something or the other. In a year, every month we have to get medicines for our children” (resident, intervention area).*

This having been said, shared problems did not necessarily lead to collective action to relieve them.

*“These small diseases like fever happen to kids while playing in filth; these fodia (skin infections) occur in head, in hands, in legs. This continuously keeps happening to someone or the other. And nobody is ready to maintain cleanliness. If one person maintains cleanliness, then four people will come running to make it dirty. This is how it is here … Here, make a wall from this side and from that side and in between, make a road. And the dustbins, the big ones that are there, keep two that side and two this side so that the filth does not happen. In this filth, the children go. How many times the mother will hit the kids saying “don’t go, don’t go, don’t go?” How much can she do to keep the child home? Firstly, the area should be clean. If the place is clean, even the kids will stay clean. Health will also be good then. Health depends on the surroundings” (resident, intervention area).*

In the face of such problems, many simply felt helpless to act and seemed to point the blame at the authorities for heaping societal problems on the same vulnerable people identified as groups by virtue of the shared, seemingly intractable, difficulties.

*“If you ask any person like me (meaning a resident of this area), then he will say that we are helpless. We stay in slums because we are helpless. (Since) the authorities here give permission to such companies, biomedical waste is brought and burnt here….Now, since 22, 30 years we have been tolerating this (garbage vehicles in our area). Due to this, there are various diseases. If you want to give all diseases in a particular area, then this is the way” (resident, area unknown).*

### Community in terms of moral status of groups

Most respondents believed that both control and intervention areas should have access to intervention services being evaluated in the cluster trial. “We feel bad that one has been given and the other has not been given … No, one should not do this. If one is giving, then give it to everyone little-little.” (resident, intervention area). Their views on fairness and study design were led by, and implicitly relied on, geographical notions of community as clusters. In one case, the trial was perceived as socially divisive unless the wider research community understood the reasons for it and the intervention might subsequently be applied to control areas.

*“How will we feel … then people will start fighting: that there it is this way, at our place there is nothing like this. Why this? This way everyone will start fighting…. I mean the ones who are understanding, they will keep quiet. Now that it has come there, then one day it will come here too: this way some people will think” (resident, intervention area).*

One respondent talked about a queue for such services and the degree of effort those in the control areas had put into the project.

*“… For example, you might say that only my lane will get the facilities that are there and that lane will not get. So what I would want is even that lane should get facilities. All the 10 areas should get … I will feel. I will put so much effort that the area in which you get more support, so that our turn may come soon … one thing that we will feel is that our turn should come soon” (resident, control area).*

Implicit in these views is the sense of humanity and moral identification with those who are denied services: “This should not happen. For both the communities, it should be the same … In a society, everyone is equal … The people there, humans are the same everywhere, aren’t they? There is no difference between people. Even they eat grains, even we eat grains” (resident, control area). A minority said that it was important to them that their area would have partial access to the services. “If my area doesn’t receive the services, then I will break Z *bhai’s* head. Because here, we people stay. We know that here such things are needed.” (resident, intervention area).

## Discussion

The term community seemed to have either a narrow or broad meaning for different respondents. For example, it was used to refer to all of Mumbai or a locality, or was understood in terms of religion (for example, all Muslims). It was also used to describe a group of people who lived in the same area and had something in common besides a shared sense of place, such as religion, ethnicity, dialect, proximity to one’s house (residents of the same lane), or a specific relationship with each other (family, relatives).

The term was never used to distinguish people of the same caste, and this raises an important issue. Communalism — in terms of conflict between identity groups — is never far from Indian consciousness and casts a shadow over politics and society. Although caste has been abolished, social divisions persist and subpopulations are still classified according to caste and tribal status. Likewise, the potential for Hindu-Muslim conflict is an ever-present cultural trope that calls to mind a history of violence that extends to the present. The Mumbai riots of 1992–1993 were largely located in poorer areas, and it may be that people felt a need to describe their communities as mutually tolerant, a counterpoint to both society’s and their own concerns.^3^Finally, the notion of the slum is often used as a means of ‘othering’ its residents and has many pejorative connotations. One could propose a scenario in which our respondents were keen to emphasize their good relations with their neighbours — particularly in terms of caste and religion — and at the same time to reframe their slum dwelling status in terms of modern urbanity, tolerance and fraternity.

About half of the respondents raised ideas of community additional to geography or locality, most of which were consistent. Some appeared inconsistent and might suggest that people felt that they belonged to more than one community, had multiple identities that either held simultaneously (as in family relations) or were drawn on singly, but were functionally dependent on the context or the question posed (for example, in response to questions about health problems or evaluation of fairness and clusters). Alternatively, they may have not had a clear view of what community is. There were no conflicting responses between individuals in the same area.

The fact that so many were sympathetic to a geographical definition of community means only that their sense of community seems compatible with the scientific notion of a cluster. Most respondents gave a geographical definition, and only two excluded their immediate neighbours. However, the boundaries of the locality, the extent of the community's reach (explicitly or implicitly identified by respondents) and the geographical areas covered by the corresponding clusters were often different. Ideas of geographical boundaries were different even within lay responses; for instance, one gave a narrow geographical definition, including only people who lived in her lane, while two others said that they considered as their community only the people who had been living in the area for many years. Only six people clearly defined their locality as their community and used phrases such as “this is a mixed community”. More than a third were prompted to give a geographical definition (most of them had given an apparently inconsistent non-geographical definition before) and eventually agreed that the people in their area — their neighbours — were also their community.

Our findings suggest that there was unlikely to be an obvious conflict between a lay and scientific view of community in the case of this particular CRT. The respondents seemed willing to agree with ideas of community, including scientific ones, once prompted, which might indicate that they were willing to internalize (or rationalize) their involvement in the trial. Some methodological limitations of the study should be noted. When respondents were asked to define their community, half of them did not understand the question to begin with, and the interviewer had to use examples. Either they were unfamiliar with the term and had not been asked to give a definition of community before, or the words that the translator was asked to use (*samaaj, basti*) and the original term ‘community’ do not have exactly the same meaning. However, by using examples and rephrasing questions we believe that responses and their translation did not bear a systematic misinterpretation of the respondents’ views.

Existing research suggests that notions of community reflect a distinct set of values and governing structures [4], as well as sufficient social interaction and permanence to allow an individual to identify herself as a community member [3]*.* Good relations between the residents of an area are not sufficient to claim that people with a shared sense of place constitute a community.^4^ In particular, some respondents said that they did not have harmonious relations with their neighbours. This might have methodological implications for cluster design and for research governance, including the choice of cluster, statistical adjustment for similarity and the roles of gatekeepers in proving cluster consultation or permission on behalf of the cluster [2].

A few people said that the reason that all residents lived in harmony, despite the fact that they lived so densely, was that they did not interfere with each other’s lives. The intervention under test encouraged community members to discuss intimate personal issues such as family planning and domestic violence. It was, therefore, important that beneficiary definitions of community were respected, in order to prevent their having to discuss this sort of information with people they did not regard as members of their group and who might not consider discretion and confidentiality important values. Responses to perceived causes of disease were also associated with geographical ideas such as environmental or living conditions and access to hospital facilities, and these resonate with the scientific notion of clusters and the need to address a shared problem. Many respondents thought that denying control clusters access to the trial intervention would be unfair, and often referred to clusters as communities, apparently adopting a scientific view and suggesting they had sympathy with the wider society of included clusters.

Future research could investigate the potential for such mismatch in other, more controversial, CRTs in order to judge whether it is an issue worthy of ethics review. The question of what a community is and how well scientists are able to incorporate such a notion seems logical prior to any analysis of balancing the social value of a CRT against the risks and potential benefits to individuals within communities. Despite the comforting findings in our study, it is still conceivable that artificial division of communities or social groups (to achieve smaller numbers of individuals in each cluster) could lead to social and political conflicts.

### Potential social disharmony and mistrust

A trial might create intra-community tensions between participants and non-participants or between intervention and control groups, linked to the provision of services and the nature of individual costs and benefits. This would undermine relations of trust and understanding between researchers and social groups who participate in research. Moreover, a potential disagreement between a lay and scientific view of community could have methodological implications, such as contamination (because of the proximity between the members of the group), and ultimately undermine the value of the study.

### Potential harms to individual members

Differences between internal and external definitions of community may also affect the interests and rights of individual members of the social groups that participate in research. Researchers usually define social groups by the way they function socially, politically or morally as whole groups, or by their genetic or disease characteristics, but the perspectives of individual members may be different. An individual’s membership may be voluntary (membership in a group may be important to an individual’s sense of identity) [25, 26], but may also be involuntary (the benefits and harms of a group may affect an individual because she has been born and raised in it and not because she has chosen it) [27]. Determining who is and who is not a member of a group can be a matter of dispute [13]. In CRTs, this dispute may be problematic, especially in the case of cluster trials that include interventions, which cannot be administered individually (cluster-cluster trials), in which individual participants cannot opt out or are not offered the opportunity to consent. Potential harms to individual members, such as stigmatization and undue influence to participate, should be considered. In contrast, individuals may not be identified by researchers as members of a social group and may be denied the right to participate.

### Uncertainty of the role of the gatekeeper

The way cluster boundaries are defined in CRTs will also affect the approach to issues of representation. A cluster might include a well-defined group — for example, a village — with legitimate political authority that could represent and protect the group’s interests and consult the researchers on group needs and values. However, a trial may include clusters with more than one well-defined group — for example, two or three villages — and offer shared resources and interventions through collaboration. If the groups have different values, needs and traditions, which cannot be reconciled, which group’s interests should take priority? How should conflicting interests (be they individual- or group-based) held by stakeholders be balanced, in theory or practice? Practical challenges concerning the resolution of disputes between different parties (especially when there is potential harm) should also be considered.

Since a variety of clusters are involved in CRTs, the degree to which group and community interests may be affected by a disagreement between scientific and lay views of community will vary. Social groups range from extremely heterogeneous to homogeneous. They may consist of geographically dispersed populations or highly localized communities that share common sociocultural traditions and whose members interact frequently [28]. Moreover, there are multiple types of relations between individuals and their groups or communities; for some individuals membership may be voluntary, and for others involuntary. Some individuals may have exclusive membership of a community, while others may be identified with several communities [29]*.* Different interests and goods will be affected in different types of communities. For instance, a trial in which cohesive communities such as villages are divided into clusters may seriously affect the maintenance and integrity of their social structures and the solidarity and unity between their members, while a trial that randomizes hospital wards would not have the same implications. In the latter case, other common interests would be at stake for cluster members (the patients in the wards), such as their interests in the quality of the services that facilities provide [13]. Finally, the degree and kinds of interests that could be affected by group participation in CRTs will also depend on the type of study. For instance, group-based interests are more likely to be substantially affected in a CRT that tests a new vaccine than in a knowledge-translation study.

### A combination of the scientific and lay approach

To provide the conceptual resources to better address potential conflicts between researchers and research participants that may be morally problematic, we wish to suggest another type of community, which incorporates/takes into account the goals/needs of both researchers and non-researcher stakeholders. This would respect residents’ needs and values and would reduce the potential methodological difficulties of defining the boundaries of the cluster and of statistically adjusting for similarities within each cluster. By using existing forums for community engagement (especially in community participatory research or community participatory action research), researchers could explore what individuals’ primary sense of community or social group comprises and, where possible, try to fit the clusters around them. The difference between this notion of community and the cluster is that it would also be based on people’s values and needs and would not be an external definition created solely for scientific purposes. It would also entail a normative process, meaning that it would presuppose that researchers know and respect the existing social relations and hierarchies and through this avoid intra-community tensions. Members of the community do not necessarily need to be close to all other members, but should be able to work together and in harmony with those who have the same health needs.

## Conclusion

Our findings suggest that, while there might be challenges to drawing cluster boundaries in CRTs, it is unlikely to be problematic in the particular trial under study. Nevertheless, we argue that it is morally and politically important to take social factors into account — as well as statistical efficiency — when choosing the size and type of clusters and designing a comparative trial. One method of informing such a design would be to use existing community forums to understand what individuals’ primary sense of community or social group comprises and, where possible, to fit the clusters around such perceptions.

## Abbreviations

CRT: cluster randomized trial
ICC: intra-cluster coefficient
NFHS: National Family Health Survey
PAD: persons of an age at risk of cardiac arrest
SNEHA: Society for Nutrition, Education and Health Action

## Footnotes

1 The 20 resource centres were opened in three phases — of 6, 6 and 8 — at six-month intervals. Each centre was rented and refurbished and its community officers recruited and trained in advance of the opening time, and all were opened and functional at the planned times. It took one year to open them all (at zero, six, and 12 months).

2 Researchers are unsure of the respondents’ location in which they lived.

3 Although we did not want to give an interpretative authority with respect to participants’ behaviours or statements, we considered that a distinction should be made between what people say about their conceptions of their community and who is in it and that they actually hold true.

4 Although good relations are more likely to lead to active cooperation.
